# The use of social network analysis in social support and care: a systematic scoping review protocol

**DOI:** 10.1186/s13643-021-01876-2

**Published:** 2022-01-10

**Authors:** Rosario Fernández-Peña, María-Antonia Ovalle-Perandones, Pilar Marqués-Sánchez, Carmen Ortego-Maté, Nestor Serrano-Fuentes

**Affiliations:** 1 Department of Nursing, University of Cantabria, Santander, Spain; 2 IDIVAL Nursing Research Group, Santander, Spain; 3 SALBIS Research Group, Leon, University of Leon, Leon, Spain; 4 Library and Information Science Department, Universidad Complutense de Madrid, Madrid, Spain; 5 NIHR ARC Wessex, School of Health Sciences, University of Southampton, Southampton, UK

**Keywords:** Health care, Scoping review protocol, Social care, Social networks, Social Network Analysis, Social support

## Abstract

**Background:**

In recent decades, the literature on Social Network Analysis and health has experienced a significant increase. Disease transmission, health behavior, organizational networks, social capital, and social support are among the different health areas where Social Network Analysis has been applied. The current epidemiological trend is characterized by a progressive increase in the population’s ageing and the incidence of long-term conditions. Thus, it seems relevant to highlight the importance of social support and care systems to guarantee the coverage of health and social needs within the context of acute illness, chronic disease, and disability for patients and their carers. Thus, the main aim is to identify, categorize, summarize, synthesize, and map existing knowledge, literature, and evidence about the use of Social Network Analysis to study social support and care in the context of illness and disability.

**Methods:**

This scoping review will be conducted following Arksey and O'Malley's framework with adaptations from Levac et al. and Joanna Briggs Institute’s methodological guidance for conducting scoping reviews. We will search the following databases (from January 2000 onwards): PubMed, MEDLINE, Web of Science Core Collection, SCOPUS, CINAHL, PsycINFO, Cochrane Database of Systematic Reviews, PROSPERO, and DARE. Complementary searches will be conducted in selected relevant journals. Only articles related to social support or care in patients or caregivers in the context of acute illnesses, disabilities or long-term conditions will be considered eligible for inclusion. Two reviewers will screen all the citations, full-text articles, and abstract the data independently. A narrative synthesis will be provided with information presented in the main text and tables.

**Discussion:**

The knowledge about the scientific evidence available in the literature, the methodological characteristics of the studies identified based on Social Network Analysis, and its main contributions will highlight the importance of health-related research's social and relational dimensions. These results will shed light on the importance of the structure and composition of social networks to provide social support and care and their impact on other health outcomes. It is anticipated that results may guide future research on network-based interventions that might be considered drivers to provide further knowledge in social support and care from a relational approach at the individual and community levels.

**Trial registration:**

Open Science Framework https://osf.io/dqkb5.

**Supplementary Information:**

The online version contains supplementary material available at 10.1186/s13643-021-01876-2.

## Background

Social Network Analysis (SNA) is a research approach within the social and behavioral sciences which focuses on ways of interaction and interconnection between individuals and social groups to explain social patterns of feelings, thoughts, and behaviors [1, 2]. In recent decades, research based on SNA has been increasingly used in health, including areas such as disease transmission, health behavior, organizational networks, social capital, and social support [3–5].

The literature on social networks and health begins by referring to the idea that people are embedded in a network of relationships. The first empirical studies were published in the Annual Review of Public Health in the mid-1990s [6]. They showed the usefulness of specific SNA techniques to evaluate prevention programs among the involved organizations [7], or the relationship between HIV status, drug use and sexual relations [8]. Since then, there has been an exponential increase in scientific publications based on this methodology, especially in the last decade.

Different SNA studies have focused on showing the relationship between the characteristics of the social network and different health-related outcomes such as health behaviors [9, 10], satisfaction with social support in chronic illness [11], quality of care and patient safety [12], the influence of social networks on HIV prevention and treatment outcomes [13], behavior change and risk of disease transmission [14], or performance in health care organizations and health care providers [15–17]. Also, SNA has been applied in health interventions based on social networks [18–21].

As mentioned above, one of the application areas of SNA is social support. The current epidemiological trend is characterized by a progressive increase in the population’s ageing and the incidence of long-term conditions. Thus, it seems relevant to highlight the importance of both social support and care systems to guarantee the coverage of health and social needs within the context of acute illness, chronic disease, and disability for patients and their careers. In its conceptual differentiation, caring and social support are dynamic processes that allude to interpersonal relationships [5, 22–24]. However, they exist predominantly in separate domains. Care belongs to the professional context, while social support refers mainly to non-professional providers [25]. Unlike other approaches, research that uses SNA to study social support and care considers the network’s structural properties as the object of study [26, 27] to know their relationship with other variables of interest. In this review, social networks are considered a structural framework to understand social support and care as relational concepts or as resources transferred through relationships [28, 29]. Since there is no previous research that synthesizes the current knowledge on this research topic, we aim to identify, categorize, summarize, synthesize, and map existing knowledge, literature, and evidence about social network analysis to study social support and care in patients or caregivers in the context of illness, disability, or long-term conditions.

## Methods

A scoping review is selected as an exploratory form of knowledge synthesis due to the extensive and growing literature that uses SNA in social support and care. This type of review is commonly undertaken to examine the extent, range, and nature of research activity in a topic area [30]: (a) to identify the types of available evidence in a given field, (b) to clarify key concepts/definitions in the literature, (c) to examine how research is conducted on a certain topic or area, (d) to identify key characteristics or factors related to a concept, (e) as a precursor to a systematic review, and (f) to identify and analyze knowledge gaps [31].

Arksey and O’Malley’s methodology framework [32], its advance by Levac and colleagues [33], and Joanna Briggs Institute’s methodological guidance [34] will be followed to conduct this scoping review through five stages: (a) identifying and stating research questions, (b) identifying relevant studies, (c) study selection, (d) charting data, and (e) collating and summarizing results [32].

This protocol is registered within the Open Science Framework platform (registration ID: https://osf.io/dqkb5 ). This scoping review has been reported using PRISMA-P [35] (Additional file 1). The final output will adhere to the Preferred Reporting for Systematic Reviews (PRISMA-ScR) checklist [36].

### Stage 1: identification of the research question

The following research questions will guide the review:

- What scientific evidence or studies are available in the literature on social support and care using the SNA methods?
- What methodological characteristics constitute this body of literature?
- What are the main contributions of these studies?
- What knowledge and research gaps can be identified in the literature?

### Stage 2: identifying relevant studies

The PCC framework (Population-Concept-Context) (Table 1) will be used to clearly define the concepts in the main review question, determine the eligibility of studies and guide the selection process [34]. We use a glossary of Terms for Community Heath Care from the World Health Organization to clarify the concepts used in our review [37].

**Table 1:** PCC framework

Population	*Patient:* a person with acute illnesses, disabilities, or long-term conditions. *Caregiver*: a person who provides support and assistance, formal or informal, through various activities to people with acute illnesses, disabilities, or long-term conditions. We consider both health professionals (nurses, medical and allied health professionals) as a formal caregiver, and personal contacts (family, friends, neighborhood, others) as informal caregivers involved in the delivery of care and social support.
Concept	*Social support:* emotional, instrumental, and financial assistance obtained from an individual's social network. Social support provided by family, friends and neighbors is referred to as informal support. In contrast, social support provided by formal service agencies is known as formal support. *Care*: the application of knowledge to the benefit of a community or individual to improve health and wellbeing.
Context	The presence of acute illnesses, disabilities, or long-term conditions in both institutional and personal settings.

The limits to be used in online databases searches will be: articles published in Spanish and English and the year of publication (from January 2000 onwards). The inclusion criteria will be (a) empirical studies with SNA methodology (quantitative or mixed methods design) and (b) studies whose participants are patients or caregivers as receivers of care or social support in the context of illness or disability provided by both, health professionals or personal/informal contacts with no age limits. The exclusion criteria will be (a) theoretical papers, (b) grey literature, and (c) qualitative studies.

The PRISMA flow chart [38] (Additional file 2) will capture and present our planned screening and selection process. The search strategy developed by MAOP will follow a comprehensive and sequential three steps and be checked by RMM. The Peer Review of Electronic Search Strategies Evidence-Based Checklist (PRESS EBC) will be followed to assess the search strategy's quality [39].

In the first step, the authors will work with an initial limited search in the PubMed database. The keywords and index terms will be identified in the titles and abstracts of the retrieved papers. In the second step, these keywords and index terms will be used to search across different databases. A structured search strategy will include Boolean operators (and, or, not), and truncations, either individually or in combination to ensure the search process. We will search the following databases: PubMed, MEDLINE, Web of Science Core Collection, SCOPUS, CINAHL, PsycINFO, Cochrane Database of Systematic Reviews, PROSPERO, and DARE (see Additional file 3 for search strategy). In a third step, a primary source search will be driven in the following journals: Social Networks, Connections, Journal of Social Structure, Redes, and Portularia. The retrieved references will be managed, and duplicates will also be removed using Mendeley and excel spreadsheet as a data extraction tool for the study selection.

### Stage 3: study selection

Titles and abstracts of identified records will be assessed by two authors (RFP and NSF), independently. Disagreements will be resolved by consensus or with the assistance of a third author (PMS). The selected studies’ full text will be retrieved and checked independently by two authors (RFP and NSF). Sources of information that do not meet the eligibility criteria will be disregarded. A record of those sources and the reasons for their exclusion will be kept in a separate file.

### Quality assessment

Scoping reviews are designed to provide an overview of the existing literature, regardless of quality. Therefore, a formal assessment of the quality of the included studies will not be conducted [32].

### Stage 4: charting the data

The data charting aims to provide a descriptive summary of the results that align with this scoping review’s research questions. Thus, a data extraction tool designed for this study has been adapted from the template data extraction instrument for scoping reviews provided for JBI Manual for evidence synthesis [34] and will be used to capture the research purpose's most relevant information (see Table 2).

**Table 2:** Initial data extraction charting template

Main category/subcategories	Description
Article data	
Author(s)	Who is/are the author(s)?
Title	The full title of the article
Publication year	When was the study published?
Country	In which country was the study conducted?
Language	What language is used in the article?
Study characteristics	
Aims/purpose	Describe the stated objective(s)
Study design	Quantitative or mixed methods. Experimental or observational
Data collection methods	Questionnaires, electronic health record extraction, online platforms network data, qualitative techniques, etc.
Key concept	Does the study focus on social support, care, or both?
Population characteristics	
Participants' role	Who are the participants? Patients, formal, or informal caregivers
Age	Participant’s age
Gender	Participant’s gender
Participant’s context	Types of acute illnesses, long-term conditions or disabilities
Setting	Where was social support or care delivered? (hospital, primary health care, self-groups, personal environment, online settings, etc.)
Sample size	How many people participated in the study?
Social network analysis methodology	
Approach	Sociocentric, egocentric or personal network approach
Social network analysis metrics	Density, centrality measures (degree, betweenness, closeness), isolates, dyads, etc.
Analysis type	Descriptive, correlation, bivariant, multivariant, or others
Data visualization	Yes/no
Software	What SNA software was used?
Knowledge contribution	
Key findings	What were the main results of the study?
Limitations	What are the main limitations of the study?

Charting the results will be an iterative process. Table 2 will be updated continuously until the end of the analysis. We will trial the extraction form on two or three sources to ensure all relevant results are extracted by at least two members of the review team [34].

### Stage 5: collating and summarizing our results

According to the data extraction template, the obtained information will be part of built evidence tables with an overall description of the papers. We will follow the Arksey and O’Malley’s methods [32] to provide a descriptive numerical analysis of the topic, including the extent, characteristics, and their distribution in the included studies. We will present specific features and outcome measures of all included studies in a diagrammatic or tabular form. A descriptive summary will accompany the tabulated and/or charted results and will describe how the results relate to the review objectives and questions. This procedure will allow identifying specific gaps in the literature that might require further research.

## Discussion

The results of this scoping review will be added to the existing review articles on the use of the SNA in the health research area as complex health care interventions [40], the behavior change [41], nursing [42], inter-organizational networks [43], or healthcare providers [44]. Specifically, this protocol describes a systematic method synthesizing the existing literature on the use of SNA to study social support and care within the context of illness and disability.

This type of review is a convenient tool to determine the coverage of the body of literature on this specific area and will give a precise indication of the number of studies available and an overview of its focus. It might be useful for uncovering emerging evidence when it is still unclear what other more explicit questions can be addressed by a more precise systematic review [45]. Thus, the broader scope and nature justify the election of a scoping review versus a traditional systematic review that would answer specific questions and require more expansive inclusion criteria [31].

The authors anticipate that this review’s results will shed light on the importance of the structure and composition of social networks to provide social support and care and their impact on other health outcomes. This differs from many studies in this topic which use non-network approaches. The knowledge about the scientific evidence available in the literature, the methodological characteristics of the studies identified based on SNA, and its main contributions will highlight the importance of health-related research’s social and relational dimensions. Furthermore, it will identify areas for future research where social networks might be considered drivers to provide further knowledge in social support and care from a relational approach at the individual and community levels. The findings of this study will be disseminated through peer-review publications and national and international conferences.

## Supplementary Information

**Additional file 1.** PRISMA-P 2015 Checklist.

**Additional file 2.** PRISMA-Flow Diagram.

**Additional file 3.** Literature search strategy.

## Data Availability

Further information related to this review can be provided upon reasonable request. Interested readers should contact the corresponding author.

## Abbreviations

SNA: Social network analysis
