# Supplementary material for: The use of social network analysis in social support and care: a systematic scoping review protocol
Source: Syst Rev. 2022 Jan 10;11:9. doi: 10.1186/s13643-021-01876-2 (PMC8751069; doi:10.1186/s13643-021-01876-2)
Supplement: Supplementary file 3 — Additional file 3. Literature search strategy. [file 13643_2021_1876_MOESM3_ESM.docx]

# Search strategy

[Search strategy 1](#_Toc89534229)

[PubMed 2](#_Toc89534230)

[Medline 3](#_Toc89534231)

[Web of Science core collection 4](#_Toc89534232)

[Scopus 5](#_Toc89534233)

[CINAHL 6](#_Toc89534234)

[PsycINFO 7](#_Toc89534235)

[Cochrane Database of Systematic Reviews 8](#_Toc89534236)

[PROSPERO 9](#_Toc89534237)

[DARE 10](#_Toc89534238)

## PubMed

| Line | Search query |
| --- | --- |
| #1 | “social network analysis” |
| #2 | “network analysis” |
| #3 | sna |
| #4 | “s.n.a.” |
| #5 | "Social Network Analysis"[Mesh] |
| #6 | 1 OR 2 OR 3 OR 4 OR 5 |
| #7 | care |
| #8 | “social support” |
| #9 | 7 OR 8 |
| #10 | 6 AND 9 |
| #11 | **limit** 10 to Spanish |
| #12 | **limit** 10 to English |
| #13 | #11 OR #12 |
| #14 | **limit** 13 to 2000- onwards |

## Medline

| Line | Search query |
| --- | --- |
| #1 | TS=("social network analysis") |
| #2 | TS=("network analysis") |
| #3 | TS=(sna) |
| #4 | TS=("s.n.a.") |
| #5 | MHX=(social network analysis) |
| #6 | 1 OR 2 OR 3 OR 4 OR 5 |
| #7 | TS=(care) |
| #8 | TS=("social support") |
| #9 | 7 OR 8 |
| #10 | 6 AND 9 |
| #11 | 10 AND Spanish (Languages) |
| #12 | 10 AND English (Languages) |
| #13 | #11 OR #12 |
| #14 | #13 AND (2020^[[1]](#footnote-1)^ or 2019 or 2018 or 2017 or 2016 or 2015 or 2014 or 2013 or 2012 or 2011 or 2010 or 2009 or 2008 or 2007 or 2006 or 2005 or 2004 or 2003 or 2002 or 2001 or 2000 (Publication Years)) |

## Web of Science core collection

| Line | Search query |
| --- | --- |
| #1 | ALL=("social network analysis") |
| #2 | ALL=("network analysis") |
| #3 | ALL=(sna) |
| #4 | ALL=("s.n.a.") |
| #5 | 1 OR 2 OR 3 OR 4 |
| #6 | ALL=(care) |
| #7 | ALL=(“social support”) |
| #8 | 6 OR 7 |
| #9 | 5 AND 8 |
| #10 | 9 AND Spanish (Languages) |
| #11 | 9 AND English (Languages) |
| #12 | #10 OR #11 |
| #13 | #12 AND (2020^[[2]](#footnote-2)^ or 2019 or 2018 or 2017 or 2016 or 2015 or 2014 or 2013 or 2012 or 2011 or 2010 or 2009 or 2008 or 2007 or 2006 or 2005 or 2004 or 2003 or 2002 or 2001 or 2000 (Publication Years)) |

## Scopus

| Line | Search query |
| --- | --- |
| #1 | TITLE-ABS-KEY ("social network analysis") |
| #2 | TITLE-ABS-KEY ("network analysis") |
| #3 | TITLE-ABS-KEY =(sna) |
| #4 | TITLE-ABS-KEY =("s.n.a.") |
| #5 | 1 OR 2 OR 3 OR 4 |
| #6 | TITLE-ABS-KEY (care) |
| #7 | TITLE-ABS-KEY ("social support") |
| #8 | 6 OR 7 |
| #9 | 5 AND 8 |
| #10 | 9 AND AND ( LIMIT-TO ( LANGUAGE , "Spanish" ) |
| #11 | 9 AND AND ( LIMIT-TO ( LANGUAGE , "English" ) |
| #12 | #10 OR #11 |
| #13 | #12 AND AND ( ^[[3]](#footnote-3)^LIMIT-TO ( PUBYEAR , 2020 ) OR LIMIT-TO ( PUBYEAR , 2019 ) OR LIMIT-TO ( PUBYEAR , 2018 ) OR LIMIT-TO ( PUBYEAR , 2017 ) OR LIMIT-TO ( PUBYEAR , 2016 ) OR LIMIT-TO ( PUBYEAR , 2015 ) OR LIMIT-TO ( PUBYEAR , 2014 ) OR LIMIT-TO ( PUBYEAR , 2013 ) OR LIMIT-TO ( PUBYEAR , 2012 ) OR LIMIT-TO ( PUBYEAR , 2011 ) OR LIMIT-TO ( PUBYEAR , 2010 ) OR LIMIT-TO ( PUBYEAR , 2009 ) OR LIMIT-TO ( PUBYEAR , 2008 ) OR LIMIT-TO ( PUBYEAR , 2007 ) OR LIMIT-TO ( PUBYEAR , 2006 ) OR LIMIT-TO ( PUBYEAR , 2005 ) OR LIMIT-TO ( PUBYEAR , 2004 ) OR LIMIT-TO ( PUBYEAR , 2003 ) OR LIMIT-TO ( PUBYEAR , 2002 ) OR LIMIT-TO ( PUBYEAR , 2001 ) OR LIMIT-TO ( PUBYEAR , 2000 ) ) |

## CINAHL

| Line | Search query |
| --- | --- |
| #1 | TI "social network analysis" OR AB "social network analysis" |
| #2 | TI "network analysis" OR AB "network analysis" |
| #3 | TI sna OR AB sna |
| #4 | TI "s.n.a." OR AB "s.n.a." |
| #5 | 1 OR 2 OR 3 OR 4 |
| #6 | TI care OR AB care |
| #7 | TI "social support" AND AB "social support" |
| #8 | 6 OR 7 |
| #9 | 5 AND 8 |
| #10 | 9 AND AND ( LANGUAGE "Spanish" ) |
| #11 | 9 AND AND ( LANGUAGE "English" ) |
| #12 | #11 AND ( LIMIT-TO PUBLICATION DATE 20000101-20211231^[[4]](#footnote-4)^) |

## PsycINFO

| Line | Search query |
| --- | --- |
| #1 | ti("social network analysis") OR ab("social network analysis") |
| #2 | ti("social network analysis") OR ab("social network analysis") |
| #3 | ti(sna) OR ab(sna) |
| #4 | ti("s.n.a.") OR ab("s.n.a.") |
| #5 | 1 OR 2 OR 3 OR 4 |
| #6 | ti(care) OR ab(care) |
| #7 | ti("social support") OR ab("social support") |
| #8 | 6 OR 7 |
| #9 | 5 AND 8 |
| #10 | 9 AND AND ( LANGUAGE "Spanish" ) |
| #11 | 9 AND AND ( LANGUAGE "English" ) |
| #12 | #10 OR #11 |
| #13 | #11 AND ( LIMIT-TO PUBLICATION DATE 20000101-20211231^[[5]](#footnote-5)^) |

## Cochrane Database of Systematic Reviews

| Line | Search terms |
| --- | --- |
| #1 | "social networks" AND care in Title Abstract Keyword |
| #2 | #1 AND PY=2000-2020 |
| #3 | "social network" AND “social support” |
| #4 | #3 AND PY=2000- onwards |

## PROSPERO

| Line | Search terms |
| --- | --- |
| #1 | Social Network Analysis |

## DARE

| Line | Search terms |
| --- | --- |
| #1 | (social network analysis OR network analysis OR sna OR s.n.a.):TI AND (care OR social support):TI FROM 2000 TO 2020^[[6]](#footnote-6)^ |
| #2 | (social network analysis OR network analysis OR sna OR s.n.a.):TI FROM 2000 TO 2020^6^ |

1. Onwards [↑](#footnote-ref-1)
2. Onwards [↑](#footnote-ref-2)
3. Onwards [↑](#footnote-ref-3)
4. Onwards [↑](#footnote-ref-4)
5. Onwards [↑](#footnote-ref-5)
6. Onwards [↑](#footnote-ref-6)
